# Detecting overlapping communities in complex networks using non-cooperative games

**DOI:** 10.1038/s41598-022-15095-9

**Published:** 2022-06-30

**Authors:** Farhad Ferdowsi, Keivan Aghababaei Samani

**Affiliations:** Department of Physics, Isfahan University of Technology, Isfahan, 84156-83111 Iran

**Keywords:** Network topology, Applied mathematics, Computational science, Information theory and computation

## Abstract

Detecting communities in complex networks is of paramount importance, and its wide range of real-life applications in various areas has caused a lot of attention to be paid to it, and many efforts have been made to have efficient and accurate algorithms for this purpose. In this paper, we proposed a non-cooperative game theoretic-based algorithm that is able to detect overlapping communities. In this algorithm, nodes are regarded as players, and communities are assumed to be groups of players with similar strategies. Our two-phase algorithm detects communities and the overlapping nodes in separate phases that, while increasing the accuracy, especially in detecting overlapping nodes, brings about higher algorithm speed. Moreover, there is no need for setting parameters regarding the size or number of communities, and the absence of any stochastic process caused this algorithm to be stable. By appropriately adjusting stop criteria, our algorithm can be categorized among those with linear time complexity, making it highly scalable for large networks. Experiments on synthetic and real-world networks demonstrate our algorithm’s good performance compared to similar algorithms in terms of detected overlapping nodes, detected communities size distribution, modularity, and normalized mutual information.

## Introduction

Complex relationships between components existing in society, technology, biology, economy, and other various fields, in many cases, can be modeled as complex networks by regarding components as nodes and relationships as edges^1^. As a consequence, all of the tools available for complex networks analysis could be applied to extract valuable information about the under investigation system. An important consideration of network structures is the possibility of classifying nodes into groups or communities^2^. Indeed, it is observed that many real-world networks have a community structure^3^. In a network, It is a crucial issue how to define communities, and its definition has to be problem-driven. Defining communities in complex networks is a multi-faceted issue that has been addressed and discussed in many studies^4^. However, according to its general definition, In a network, community refers to a group of nodes that are densely connected internally and have a sparser connection with the rest of the network^3^. Detecting communities is of great importance since nodes in a community usually have similarities in function, property, and characteristics^5^. For instance, community detection in the network of protein-protein interaction could reveal groups of closely connected proteins that possess an identical function in the body^6^. The discovery of community structure can be constructive in many fields, such as drug discovery^7^, precision marketing^8^, brain neural network^9^, online social interaction analysis^10^, and public opinion analysis^11^. Network communities typically can be categorized into two types. Disjoint communities with no shared members (also called non-overlapping communities or partitions) and overlapping communities with shared members (also called covers). Examples of overlapping communities are widely seen in the real world. Researchers, based on their various research interests or multiple affiliations, can be a member of more than one research group, or a gene can be involved in causing various diseases^12^. As a result, it is crucial to design community detection algorithms that be able to identify overlapping nodes. In recent years, a variety of approaches, including greedy algorithms based on modularity optimization^3,13^, label propagation algorithms (LPA)^14^, Stochastic block models^15^, and Edge betweenness algorithms, have been employed for this purpose^13^.

The use of game theory in this context was initialized by Athey and Jha in 2006 to model an organization's workers interaction^16^ and followed by a game theoretic-based algorithm proposed by Chen in 2010^17,18^. A comprehensive discussion of game theoretic-based methods for detecting community structure in networks is provided in a survey done by Jonnalagadda and Kuppusamy^18^. However, the number of game theoretic based algorithms proposed in the last decay is not very large, and most of them are not scalable for large networks^12^. Community detection algorithms using the game theory are typically based on cooperative or non-cooperative games. Our proposed algorithm is based on the non-cooperative game in which nodes are assumed as rational selfish players who decide to be part of the communities which bring them the most profit. Although our algorithm is designed to detect overlapping communities, in contrast with similar algorithms, nodes are not allowed to be part of multiple communities before the exact boundaries of communities are determined (phase one), and overlapping nodes are identified in phase two. Such two phases algorithm not only increase accuracy but also, along with the appropriate stop criterion used in current work, speed up convergence. Moreover, in the present work, players have only local interactions, which leads the algorithm to be more effective than some other game theoretic-based algorithms in which interaction with all nodes is considered in the utility function^17,19,20^. The remainder of this paper is organized as follows. In the next section, the framework of the proposed algorithm and related definitions are given, and it is followed by a discussion on the time complexity of the algorithm. Afterward, the experimental results of our algorithm and its comparison with some other state-of-the-art algorithms are given. Finally, the concluding remark is stated.

## Proposed algorithm

The proposed algorithm consists of two phases. The non-cooperative game is the basis of the first phase leading to non-overlapping community detection, while in the second phase, the overlap of the communities is determined. The game-theoretic framework is based on considering each node as a selfish agent trying to maximize its payoff by choosing different strategies, and each agent’s choice can influence the other ones’. Strategy is a term in game theory that in the current context, refers to the communities in which the agent wants to participate. Based on this, each agent’s strategy $s_i$ is actually a list of community labels it is a member of, and the strategy profile of all agents is defined as $S=(s_1,s_2, \ldots ,s_n )$. As stated, each agent aims to maximize its payoff, which for the agent $v_i$ is represented through a utility function defined as follows.

1 $$\begin{aligned} U(S_{-i},s_i)=\sum _{a_{ij} \ne 1}(1+sim_{ij})\frac{|s_i \cap s_j|}{\sqrt{|s_j|}} \end{aligned}$$

Where $a_{ij}$ is the adjacency matrix element; $s_i$ is the strategy of agent $v_i$, and $S_{-i}$ is the strategy profile of all agents but her; $|s_i \cap s_j|$ is the number of common labels between agent $v_i$ and $v_j$; and $|s_j|$ is the number of communities agent $v_j$ belongs to. Unlike some other game-theoretic overlapping community detection algorithms, in phase one agents are not allowed to acquire multiple labels and consequently, expression $\frac{(|s_i \cap s_j |)}{(|s_j |)}$ can only have two values of 0 or 1. Also, in this phase agents are only allowed to do switch operation among different community labels. In utility function, $sim_{ij}$ is the similarity between agents $v_i$ and $v_j$, which can be calculated through different available metrics as follows.

2 $$\begin{aligned} Jaccard \,\, coefficient (JC): sim_{ij}^{JC}= & {} \frac{|\Gamma _i \cap \Gamma _j|}{|\Gamma _i \cup \Gamma _j|} \end{aligned}$$

3 $$\begin{aligned} Saltin \,\, index \, (SI): sim_{ij}^{SI}= & {} \frac{|\Gamma _i \cap \Gamma _j|}{\sqrt{|\Gamma _i||\Gamma _j|}} \end{aligned}$$

4 $$\begin{aligned} Sorensen \,\, index \, (SO): sim_{ij}^{SO}= & {} \frac{2|\Gamma _i \cap \Gamma _j|}{|\Gamma _i|+| \Gamma _j|} \end{aligned}$$

5 $$\begin{aligned} Hub \,\, promoted \,\, index \, (HP): sim_{ij}^{HP}= & {} \frac{|\Gamma _i \cap \Gamma _j|}{min(|\Gamma _i|,|\Gamma _j|)} \end{aligned}$$

6 $$\begin{aligned} Hub \,\, depressed \,\, index \, (HD): sim_{ij}^{HD}= & {} \frac{|\Gamma _i \cap \Gamma _j|}{max(|\Gamma _i|,|\Gamma _j|)} \end{aligned}$$

**Figure 1:**
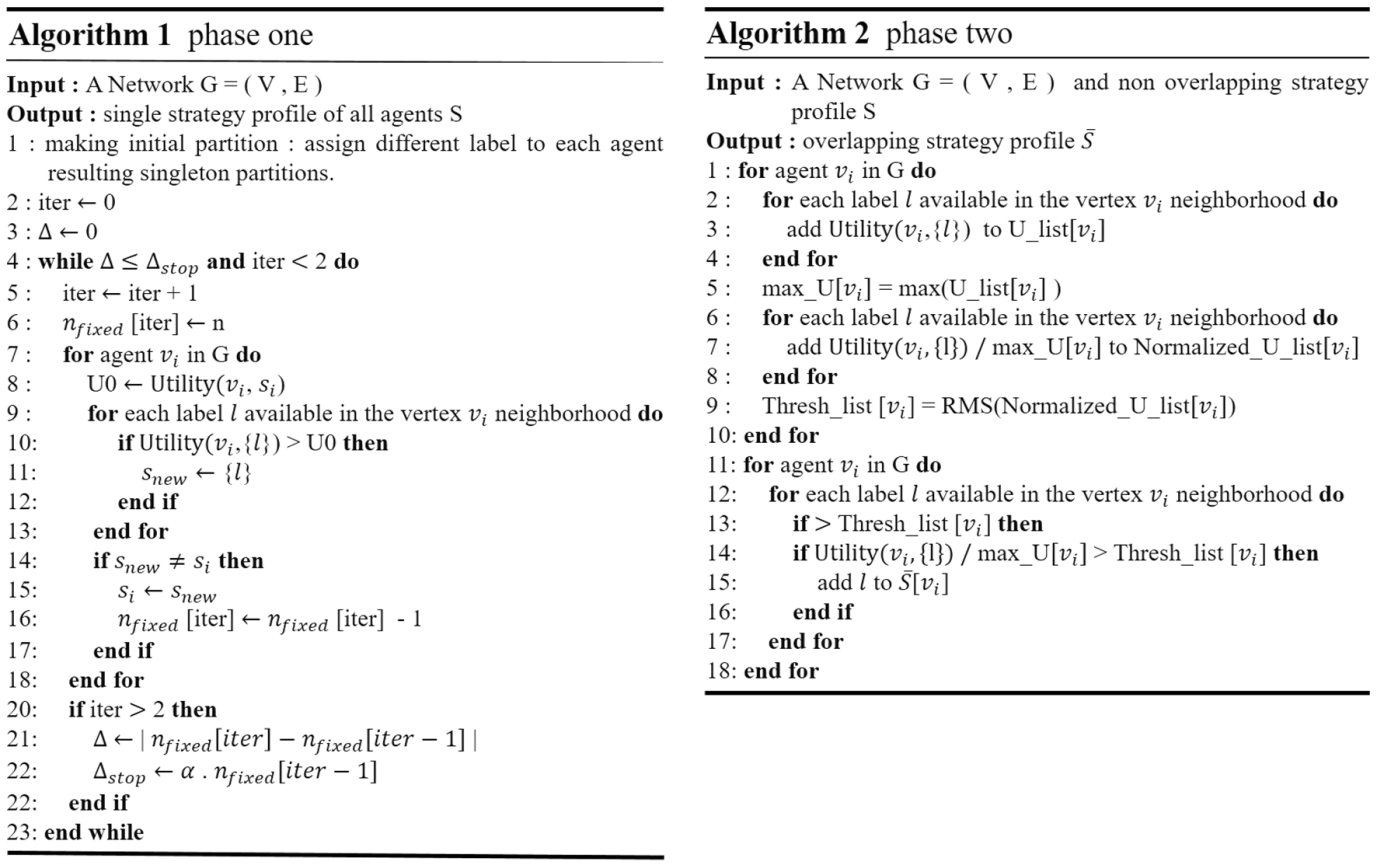
Phase one and Phase two framework algorithms.

Where $\Gamma _i$ and $\Gamma _j$ denote the neighbors of agents $v_i$ and $v_j$ , respectively. The proposed algorithm results do not significantly depend on different similarity metrics except for a few special cases. However, represented results have been obtained using HP similarity, which slightly performs better than other similarity metrics. The algorithm starts with an initial condition in which each agent $v_i$ is assigned to a singleton community $c_i$. Next, in each iteration, all agents, by order of their degrees, update their strategy by imitating their neighbors with the aim of maximizing payoff. For more clarity, the phase one framework is given in Algorithm 1 in Fig. 1.

Lines 4 to 23 repeat until the stop criterion is met and finally agents with the same label belong to the same community. The stop criterion should be defined in a way that satisfies accuracy and efficiency at the same time. In the proposed algorithm, there are some cases in which some agents’ strategy fluctuates permanently, and some other agents need too many iterations to reach their stable one. Since a minimal number of agents often fall in such category, defining a stop criterion that ignores such agents' stability could speed up the algorithm without significant loss of accuracy. For this reason, instead of waiting for all agents’ strategy to be fixed, the stop criterion is satisfied as soon as the number of agents with a fixed strategy does not increase more than a specific value. This value in each iteration $\Delta _{stop}$ is defined as a fraction of fixed agents number $n_{fixed}$ in the previous iteration.

7 $$\begin{aligned} \Delta _{stop}=\epsilon .n_{fixed} \end{aligned}$$

**Figure 2:**
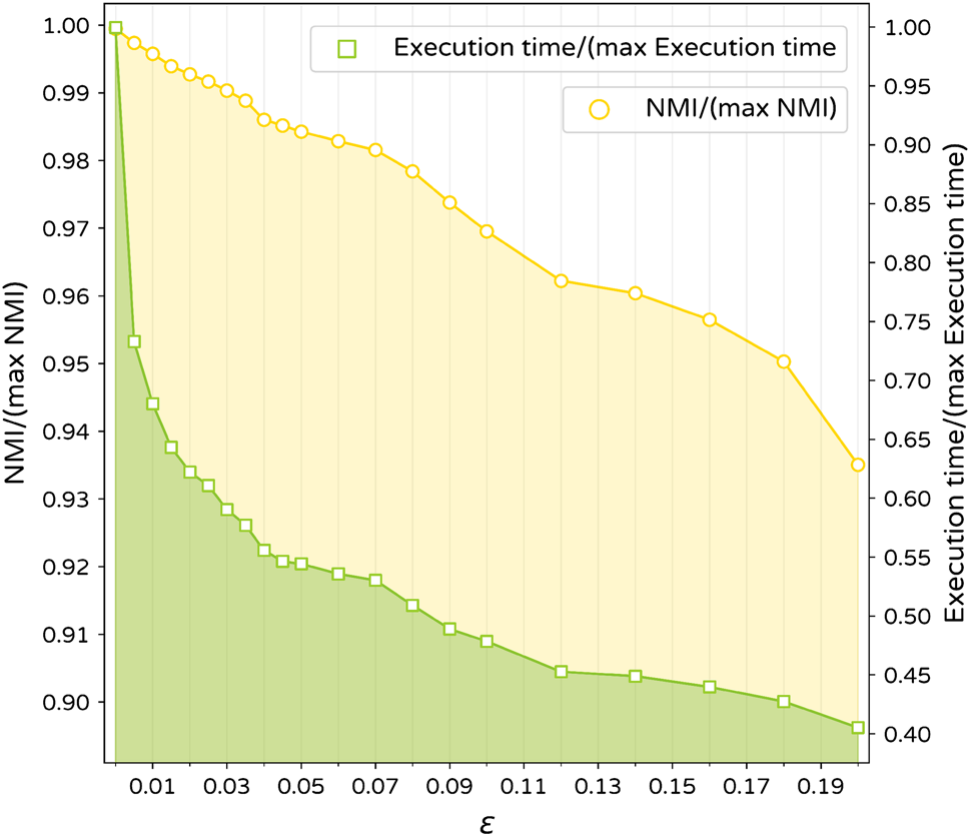
Effect of $\epsilon$ value on algorithm accuracy and elapsed time.

By adjusting $\epsilon$ value, a balance between accuracy and efficiency can be obtained. Variation of relative phase one execution time (execution time divided by longest execution time) and relative NMI (Obtained NMI divided by the best achievable NMI) obtained for LFR synthetic networks is represented in Fig. 2. According to the results, nonzero but small values of $\epsilon$ such as 0.005 and 0.01 can reduce phase one elapsed time while giving acceptable accuracy. The effect of $\epsilon$ value on the scalability of the algorithm will be discussed more in the algorithm complexity section.

**Figure 3:**
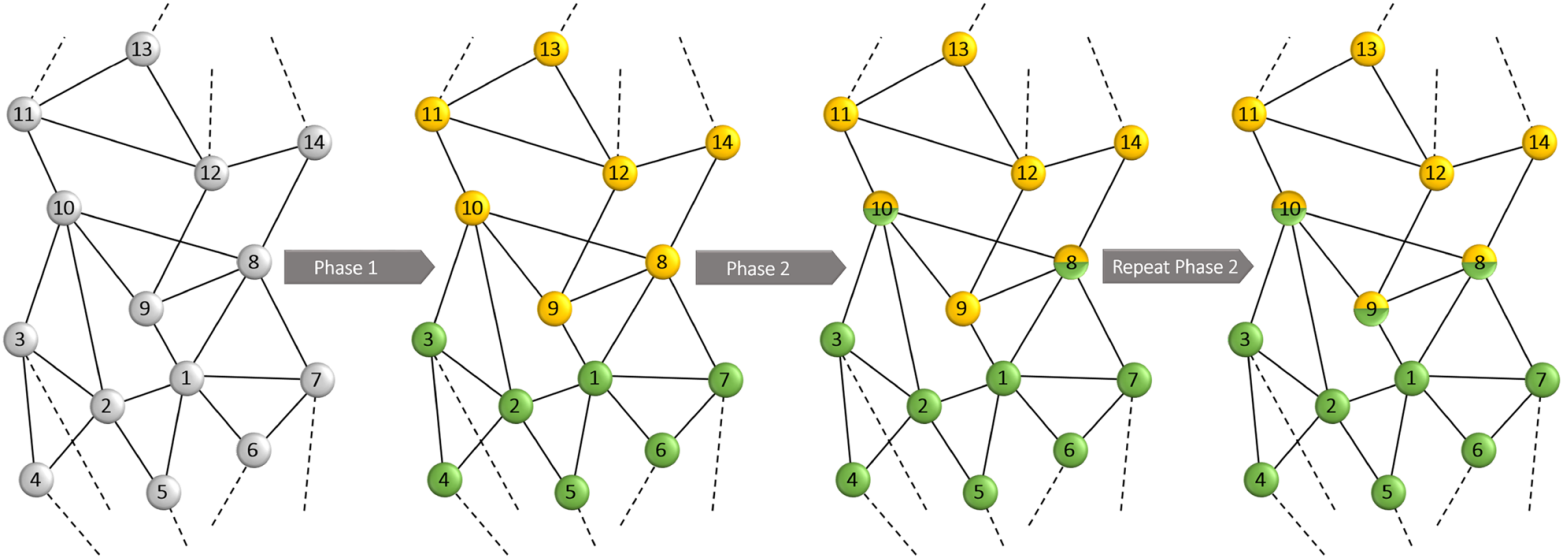
The toy example to illustrate the performance of each phase.

Phase two is responsible for finding overlapping nodes. In some non-cooperative game-theoretic algorithms, a *loss* *function* is used as a method for controlling multiple memberships of agents^17,19–21^. In such a method multiple membership criteria usually are defined in a way that is similar for all nodes in spite of different conditions they may have. Moreover, in some other algorithms like^22,23^, the manually defined threshold is responsible for determining multiple memberships of nodes. Nevertheless, in our algorithm, this criterion is defined uniquely for each agent based on payoffs it acquires from membership in different communities. Accordingly, phase two contains two stages. In the first stage in which payoff thresholds are calculated, the following operations should be done for each agent:

1. Calculating payoffs that agent acquires by Adopting any of community label available in its neighborhood.
2. Normalizing all obtained payoffs with respect to maximum payoff the agent has obtained.
3. Finding payoff threshold for the agent by calculating root mean square of normalized payoff values obtained for that agent.

In the following stage, each agent adds community labels that have a payoff above her payoff threshold value to her strategy. The framework of phase two is given in Algorithm 2 in Fig. 1.

Finally, each agent belongs to all communities which those labels exist in its strategy list. In networks with a high degree of overlap, it is very probable for overlapping nodes to be connected with other overlapping nodes. In such cases, repetition of phase two can help discover overlapping nodes more reliably. For more illustration, a toy model representing community structure before and after applying each phase is shown in Fig. 3.

It should be noted that described phase two returns crisp communities with binary membership coefficient of nodes in different communities. Although often it is the desirable form, sometimes the fuzzy communities are more suitable for the intended use. In such cases, the normalized payoff values of each agent are representative of that agent's fuzzy membership coefficients.

## Time complexity of algorithm

**Figure 4:**
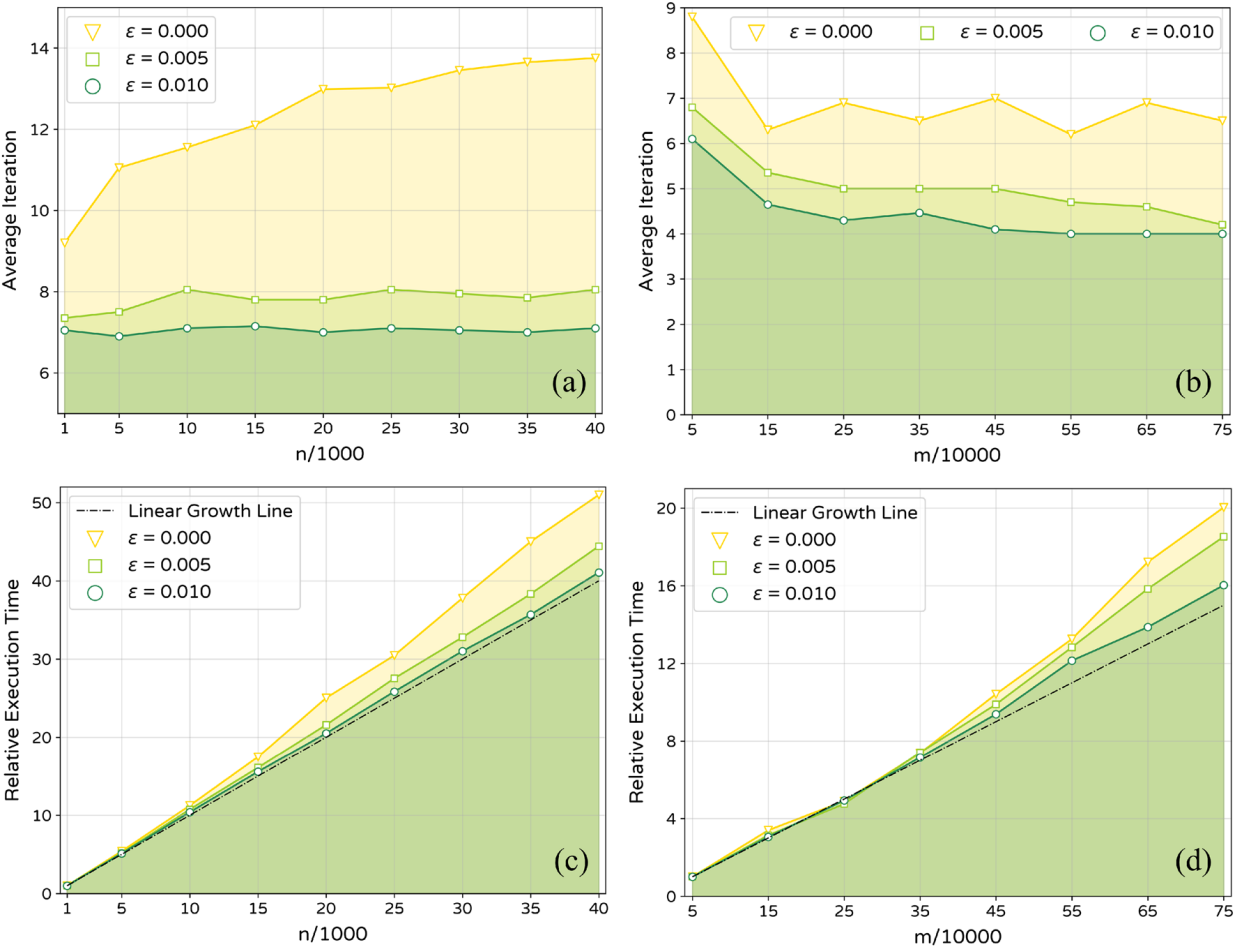
(**a**, **b**) Phase one average max iteration as a function of *n* and *m*. (**c**, **d**) Entire relative algorithm execution time as a function of *n* and *m*. Results of a and c is obtained for LFR networks with ${\overline{k}}=10$. Results of b and d is obtained for LFR networks with $n=5000$.

The proposed algorithm consists of three parts. The first one is initialization which requires *O*(*n*), where n is the total number of nodes. In phase one, the outer loop continues until stop criterion satisfaction. In inner loops, for each agent, the payoff should be calculated for all labels in the neighborhood, which is maximally equal to the number of the agent’s neighbors. Therefore, phase one requires *O*(*T*.*n*.*K*) on average, where *K* is the average degree and *T* is the maximum iteration. In some other algorithms, *T* is defined manually. In the proposed algorithm, although the maximum iteration number is determined dynamically based on stop criterion satisfaction, it does not depend on *n* or the total number of edges *m* if the network topology is kept the same and if the $\epsilon$ value is selected appropriately. For LFR synthetic networks, the variation of the maximum iteration number for three small values of $\epsilon$ were calculated as a function of *n* and *m* (Fig. 4a,b). As it can be seen, especially for small nonzero values of $\epsilon$ the maximum iteration number does not depend considerably on *n* or *m*. Phase two has a similar calculation structure as the inner part of the phase one algorithm. Considering the second phase repeats two times, it requires *O*(2*n*). Therefore, the time complexity of the entire algorithm is *O*(*n*) in sparse networks and *O*(*m*) in arbitrary ones. For a naive implementation of the algorithm, Fig. 4c,d shows the execution time for LFR synthetic networks. As it can be seen, for $\epsilon$ value of 0.01, the execution time is just slightly slower than linear growth.

## Experimental results and comparison

With the aim of evaluating our proposed algorithm performance, we compare it with some other algorithms named GAME1^17^, GAME2^24^, GAME3^25^, SLPA^22^, OSLOM^26^, CPM^27^, GCE^28^ and LFM^29^. GAME1 is based on non-cooperative game theory with the time complexity of $O(m^2)$. GAME 2 and GAME3 are based on cooperative game theory with the time complexity of $O(n^2)$ and $O(n.log(n) )+O(n.k_{max})$, respectively ($k_{max}$ is graph maximum degree). Our algorithm results in this section are obtained by set $\epsilon$ value to 0.01. Other algorithms' results are extracted from those algorithms' original papers or comparative study papers^30^. In these papers for algorithms with tunable parameters, it is stated that the results with the best setting are reported.

### Evaluation criteria

There are various metrics in order to evaluate obtained results of algorithms, and it is often challenging since no canonical solutions are available^31^. A comprehensive discussion about the relationship between the topological properties of the community structure and the alternative evaluation measures and reliability of different evaluation criteria has been addressed in many studies^32^. In the first place, choosing appropriate evaluation criteria depends on whether there is known ground truth for the examined network. In the cases with known ground truth, different evaluation measures, including Average F1 score (AvgF1)^33^, Adjusted Rand Index (ARI), which ensures that the value of random clustering is close to zero, Omega Index^34^, which is the overlapping version of ARI^30^ and adopts the number of clusters that each pair of nodes shares, to compare the detected communities versus ground truth communities, and Normalized Mutual Information (NMI)^35^, derived from information theory, are widely used. In the current work, we used AvgF1 and an extended version of NMI, which is appropriate for comparison of two overlapping community structures^29^. The closer value of NMI or AvgF1 to 1, the more similar the detected community structure to ground truth; and the 0 value indicates the least similarity.

When it comes to testing the performance of overlapping community detection algorithms, especially when the ground truth of communities is unknown, the $Q_{ov}$ is a well-known and frequently used metric^36^. It is an extension of the classical modularity, and the higher value of this means the better-detected communities. For directed networks this metric is defined as follows:

8 $$\begin{aligned} Q_{ov}=\frac{1}{m} \sum _{c \in C} \; \sum _{i,j \in V} \left[ \beta _{l(i,j),c}A_{j,j}-\frac{\beta _{l(i,j)}^{out}k_{i}^{out}\beta _{l(i,j)}^{in}k_{j}^{in}}{m} \right] \end{aligned}$$

By applying minor changes as follows, it can be used for undirected networks:

9 $$\begin{aligned} Q_{ov}=\frac{1}{2m} \sum _{c \in C} \; \sum _{i,j \in V} \left[ \beta _{l(i,j),c}A_{j,j}-\frac{\beta _{l(i,j)}^{'}k_{i}\beta _{l(i,j)}^{'}k_{j}}{2m} \right] \end{aligned}$$

The components of this equation is given by:

10 $$\begin{aligned} \beta _{l(i,j)}^{'}=\beta _{l(i,j)}^{out}= & {} \beta _{l(i,j)}^{in}= \frac{\sum _{i \in V}F(\alpha _{i,c},\alpha _{j,c})}{|V|} \end{aligned}$$

11 $$\begin{aligned} \beta _{l(i,j)}= & {} F(\alpha _{i,c},\alpha _{j,c}) \end{aligned}$$

12 $$\begin{aligned} k_{i}^{out}=k_{i}^{in}= & {} k_{i} \end{aligned}$$

13 $$\begin{aligned} F(\alpha _{i,c},\alpha _{j,c})= & {} \frac{1}{(1+e^{f(\alpha _{i,c})})(1+e^{f(\alpha _{j,c})})} \end{aligned}$$

14 $$\begin{aligned} f(x)=2px-p, p \in {\mathbb {R}} \end{aligned}$$

where $\alpha _{(i,c)}$ is the belonging coefficient of node *i* to community *c* and p in *f*(*x*) is an arbitrary value that in the current study is set to 30.

### Synthetic networks

**Figure 5:**
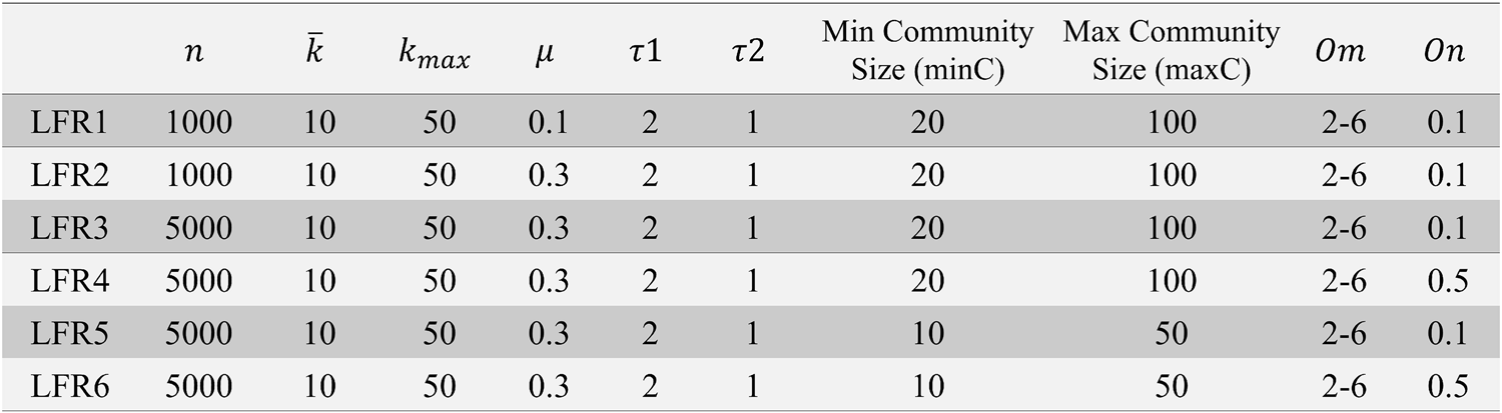
LFR synthetic networks used for performance tests.

**Figure 6:**
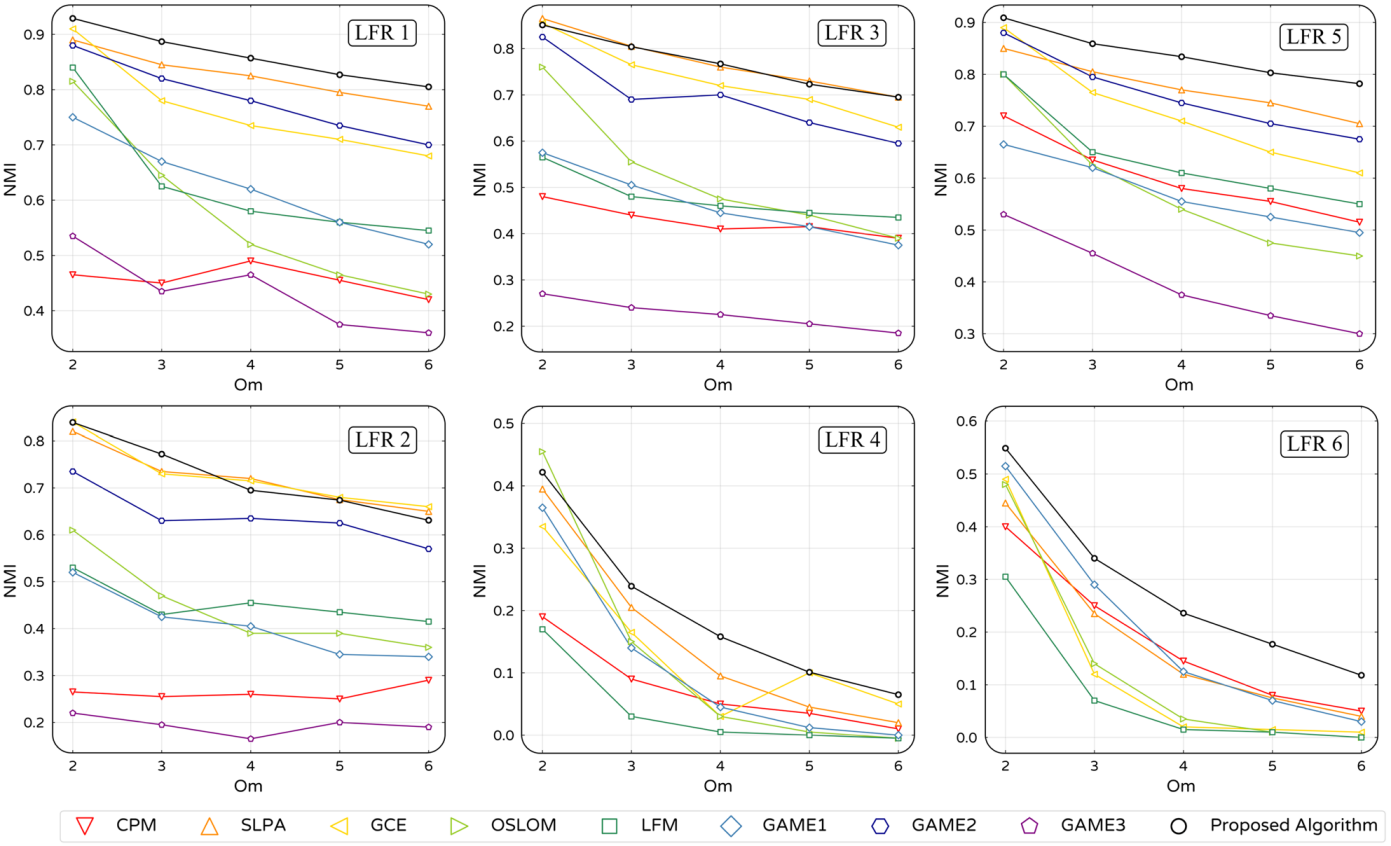
Comparative NMI value for proposed and other algorithms on LFR synthetic networks listed in Fig. 5.

One of the most famous benchmark networks is synthetic networks called LFR which can be generated by the method proposed by Lancichinetti and Fortunato^37^. While in real-world networks, degree correlation among nodes is clearly nonzero, and the transitivity is relatively high, networks generated by LFR method have near-zero degree correlation and low transitivity^38–40^. Despite this drawback and some other limitations of LFR method, these networks still exhibit relatively very high realistic properties, and considering a large amount of experimental data available from the test of other algorithms on them, LFR networks are among the most proper choices for community detection algorithms performance test. In the networks made by this method 10 parameters are adjustable. By setting these parameters, we generated 6 groups of LFR networks for the performance tests, as shown in Fig. 5. The mixing parameter $\mu$ refers to the fraction of links through which a node connects to other nodes in other communities; $k_i^{in}=(1-\mu )k_i$. $\tau _1$ and $\tau _2$ are exponents of power-law distribution of node degrees and community sizes, respectively. Furthermore, overlapping features of LFR network are controlled by *Om* (the number of communities to which each overlapping node belongs) and *On* (the fraction of nodes that belongs to more than one community). It should be noted that for our algorithm performance test on LFR networks, we have reported averaged results of runs over at least 10 instantiations of these networks for each parameter set.

The NMI values for results obtained using our proposed and other algorithms are represented in Fig. 6. As expected, by increasing *Om,* the NMI values gradually decrease. However, it is observed that in most cases, our algorithm outperforms others, especially in synthetic networks with smaller community sizes and more overlapping nodes.

**Figure 7:**
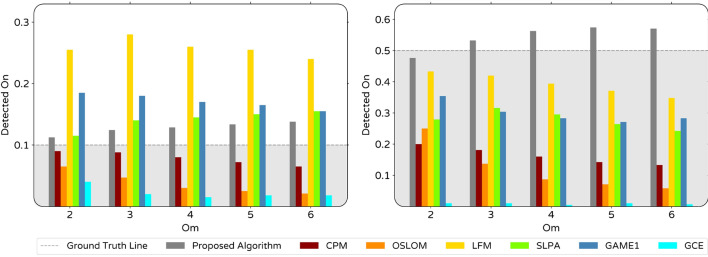
Overlapping nodes fraction detected by proposed and other algorithms in LFR3 and LFR4.

**Figure 8:**
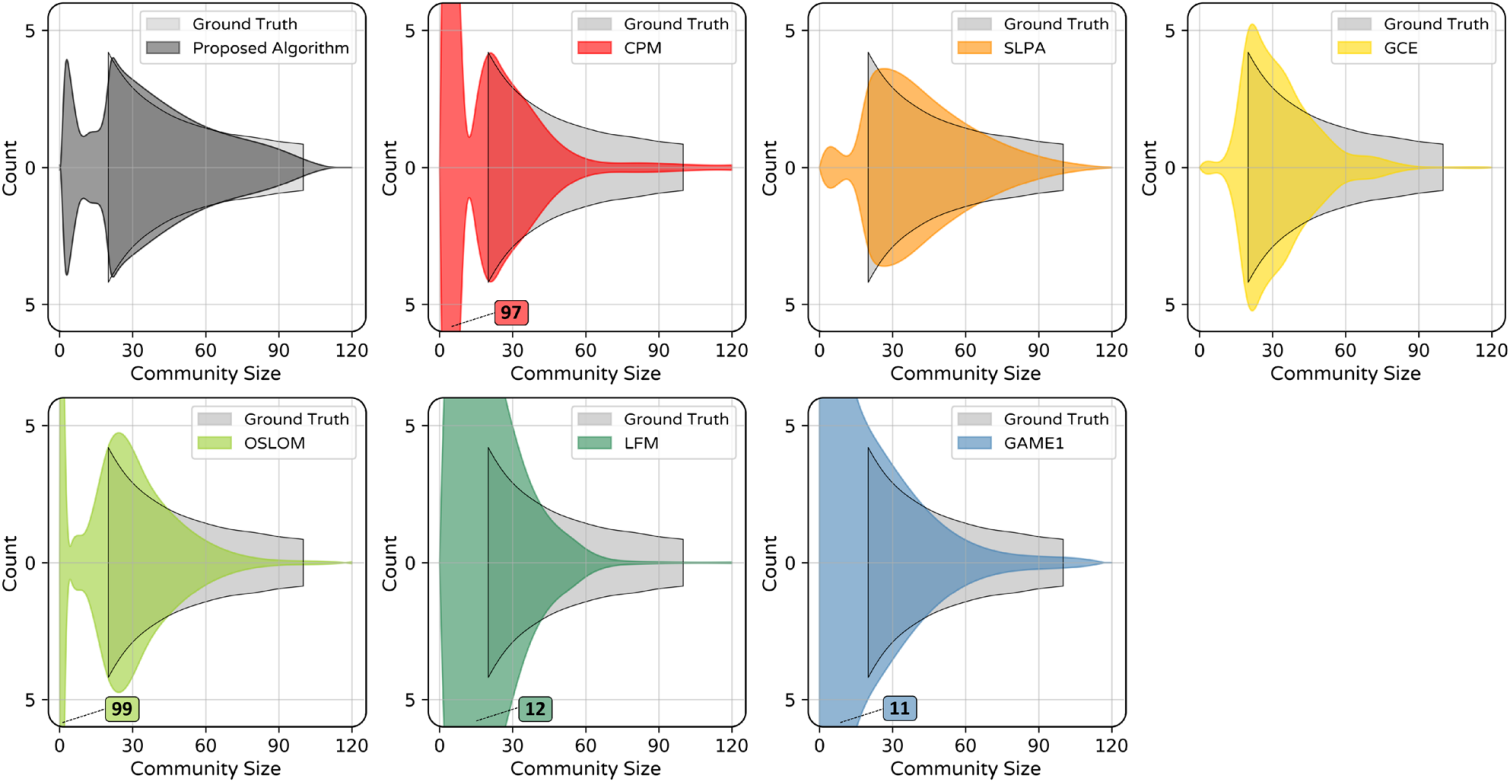
Histogram of detected community sizes for LFR3 (averaged on all *Om*). In each plot, the height of peaks is written next to them if they locate out of the frame.

When it comes to networks with overlapping communities, evaluation of a community detection algorithm performance must include checking the number of identified overlapping nodes, which is one of the important parameters determining the algorithm’s accuracy. Overlapping nodes play a crucial role in real-world social networks considering the fact they usually act as bridges or messengers between communities^30^. Identified On detected by proposed and other algorithms for two groups of LFRs with ground truth On of 0.1 and 0.5 are shown in Fig. 7. Overlapping nodes identified by our algorithm increase gradually by the increase of *Om*. This trend is in contrast with other algorithms except SLPA in LFR3 network.

Aiming to find more comprehensive insight into algorithms performance, it would be beneficial to investigate the distribution of detected community sizes (*CS*). For this purpose, we used algorithms results on LFR3 averaging on all values *Om* and 10 instantiations of these networks. In the histogram of community sizes which is shown in Fig. 8, small fluctuations were omitted by representing fitted curves instead of raw data. For comparison, the ground truth power law distribution is visible in each histogram. Except for ours and SLPA algorithms, other algorithms have remarkable weaknesses in detecting larger size communities. Besides, some algorithms tend to break communities into smaller parts that cause distribution concentration in the range of small communities which do not exist in real distribution. Although such miss clustering occurs to some extent by our algorithm, it is not as much as some other algorithms such as GAME1, LFM, and especially CPM and OSLOM. Particularly, results demonstrate the relatively better performance of our algorithm in detecting larger communities.

### Real networks

**Figure 9:**
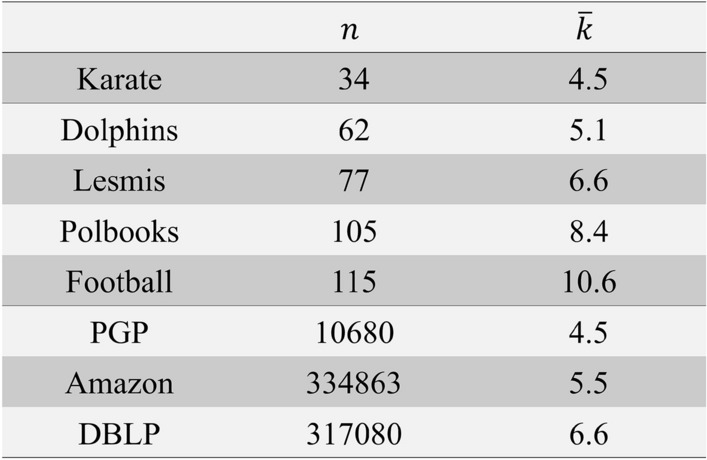
Real networks in test.

**Figure 10:**
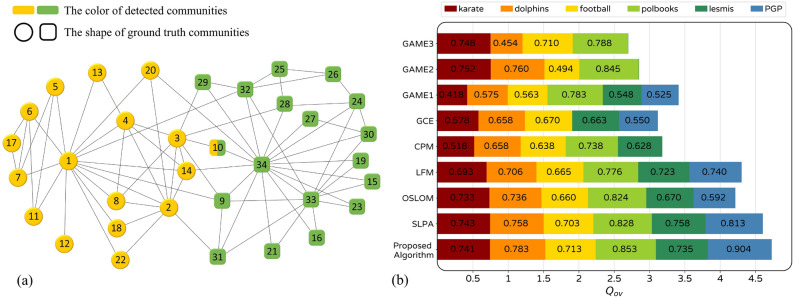
(**a**) Ground truth and detected community structure of karate network. (**b**) The $Q_{ov}$ value obtained by proposed and other algorithms on first six real-world networks.

**Figure 11:**
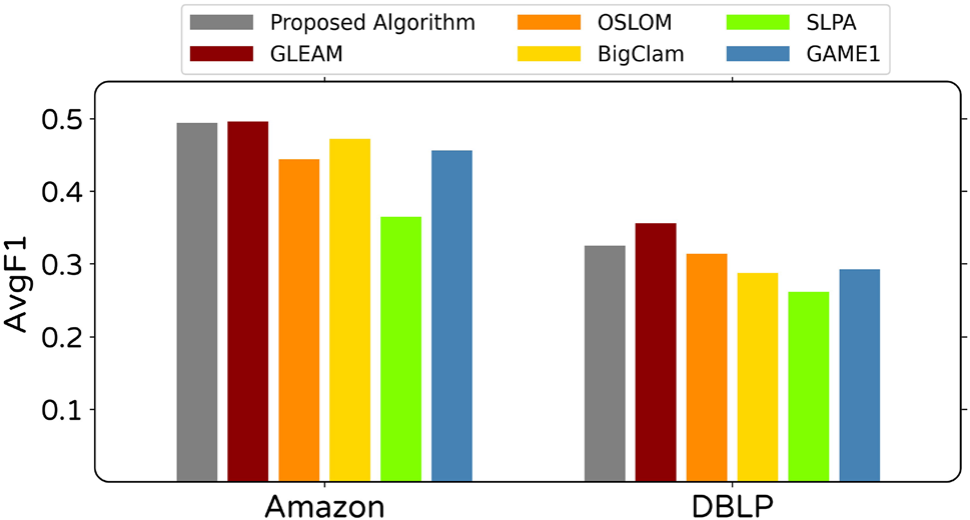
The AvgF1 score value obtained by the proposed and other algorithms on last two real-world networks with known community structure (ground truth).

In order to further evaluation of the proposed algorithm, we tested its performance on some real-world networks. Eight real networks have been chosen for this test, and their description can be observed in Fig. 9 (Data for the last three larger networks are available at http://snap.stanford.edu). As an evaluation measure, for the first six networks and for the last two ones, the overlapping modularity and AvgF1 score were used, respectively.

Stack bar chart of $Q_{ov}$ for obtained community structure of first six networks by ours and other algorithms are shown in Fig. 10. Such illustration makes us able to compare the overall performance of algorithms on all six networks. Our algorithm gets $Q_{ov}$ value for Dolphins, Football, Polbooks, and PGP, which is slightly higher than other algorithms. Moreover, the sum of $Q_{ov}$ obtained by our algorithm is higher than the others. As an example, the community structure of the karate network, which is obtained by our algorithm, is shown in Fig. 10. This network is of traditional importance and was studied by Wayne W. Zachary for three years, from 1970 to 1972^41^. The ground truth of this network that was observed by Zachary contains two communities represented in Fig. 10. As it can be seen, the detected community structure is exactly fitted to ground truth if excluding node 10. However, locating node 10 in the overlapping of two communities is sensible, considering its equal connection with both.

For the last two larger networks, which have know community structure, the bar chart of AvgF1 scores for obtained community structure by ours and other algorithms are shown in Fig. 11. For these networks, in addition to previously used algorithms, the result of BigClam^33^ and GLEAM^5^ algorithms are represented for comparison. Data related to other algorithms’ performance on these two networks are extracted from GLEAM algorithm’s original paper^5^. Based on the results represented in 11, it can be seen that the proposed algorithm, along with the GLEMAo algorithm, has the best performance in the detecting community structure of these two networks.

## Conclusion

In this paper, we proposed a novel game theoretic-based algorithm for community detection in networks. The algorithm performance test on synthetic and real-world networks indicates our algorithm has a relatively better performance compared with similar algorithms presented in the literature. Our proposed algorithm has a time complexity of *O*(*m*), making it a good choice for applying on ultra-large networks. Besides, no stochastic factors are influencing the process of community detection, which eliminates the need for multiple executions and averaging of results and causes our algorithm to be categorized among stable ones. In addition, this framework can be straightforwardly applied to weighted networks by making minor changes.

## Supplementary Information

Supplementary Information.

## Data Availability

All data generated or analyzed during this study are included in this published article. The proposed algorithm python code is available in the Supplementary Material.
